# Supplementary material for: Predicting visual function by interpreting a neuronal wiring diagram
Source: Nature. 2024 Oct 2;634(8032):113–23. doi: 10.1038/s41586-024-07953-5 (PMC11446822; doi:10.1038/s41586-024-07953-5)
Supplement: Supplementary file 6 — CRF and ERF predictions for individual TmY4 and TmY9 cells. Analogous to Supplementary Data 3, but for TmY target types. Shown are the top four monosynaptic pathways, the strongest pathway passing through each of the top ten intermediary types (ranking from Extended Data Fig. 7), and the trisynaptic pathway Tm1–TmY–Dm3–TmY (see the section entitled Prediction of spatial normalization). [file 41586_2024_7953_MOESM6_ESM.zip › DataS4/TmY9q/720575940635062196.pdf]

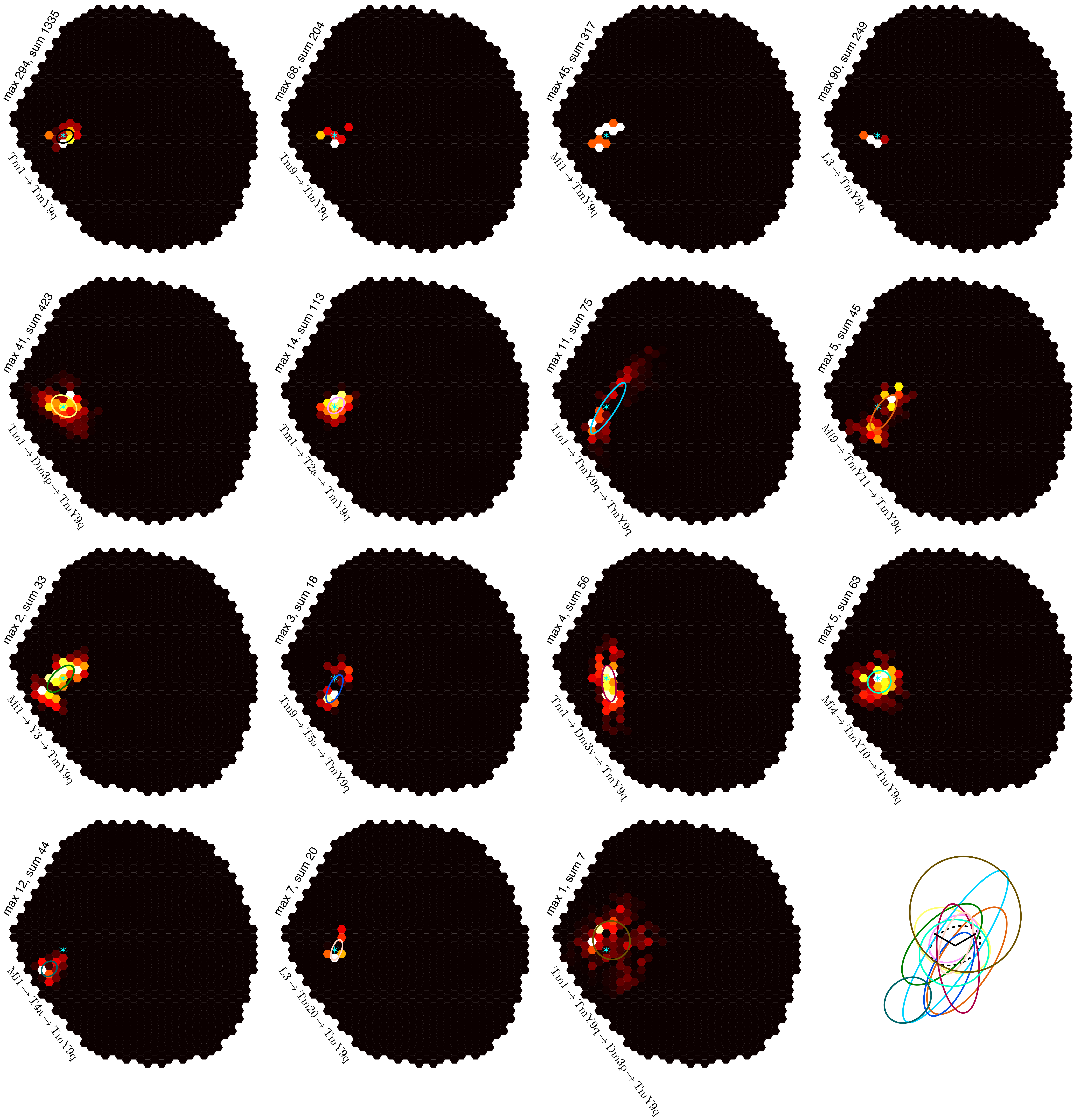

max 294, sum 1335
Tm1 → TmY9q
max 68, sum 204
Tm9 → TmY9q
max 45, sum 317
Mi1 → TmY9q
max 90, sum 249
L3 → TmY9q
max 41, sum 423
Tm1 → Dm3p → TmY9q
max 14, sum 113
Tm1 → T2a → TmY9q
max 11, sum 75
Tm1 → TmY9q → TmY9q
max 5, sum 45
Mi9 → TmY11 → TmY9q
max 2, sum 33
Mi1 → Y3 → TmY9q
max 3, sum 18
Tm9 → T5a → TmY9q
max 4, sum 56
Tm1 → Dm3v → TmY9q
max 5, sum 63
Mi4 → TmY10 → TmY9q
max 12, sum 44
Mi1 → T4a → TmY9q
max 7, sum 20
L3 → Tm20 → TmY9q
max 1, sum 7
Tm1 → TmY9q → Dm3p → TmY9q
